# Supplementary material for: The Profile of MicroRNA Expression in Bone Marrow in Non-Hodgkin’s Lymphomas
Source: Diagnostics (Basel). 2022 Mar 3;12(3):629. doi: 10.3390/diagnostics12030629 (PMC8947746; doi:10.3390/diagnostics12030629)
Supplement: Supplementary file 1 [file diagnostics-12-00629-s001.zip › diagnostics-1574903 Supplementary/Supplementary Table S2.pdf]

Table S2. The sequences of primers and miRNA probes.

Target	Type	Sequence (5′ → 3′)
miR-10b-5p	RT primer	GTCAGAGCTGAGGCTCACTGAGACCTTTCGCACCCTCGCTCTGACCACAA[illegible]
	Reverse PCR primer	CTGAGGCTCACTGAGACCT
	PCR probe	(SIMA)-TTCGCACCC(T-BHQ1)CGCTCTGACCACAAATTCG
	Forward PCR primer	ACCACTACCCTGTAGAACCG
miR-103a-3p	RT primer	GTCGTGTCTGAGGCTCACTGAGACCTATTCGCACCCTCGACACGACTCAT[illegible]
	Reverse PCR primer	CTGAGGCTCACTGAGACCT
	PCR probe	(SIMA)-ATTCGCACCC(T-BHQ1)CGACACGACTCATAGCC
	Forward PCR primer	CAGCAGCAGCATTGTACAG
miR-124-3p	RT primer	GTCGTGTCTGAGGCTCACTGAGACCTATTCGCACCTCGACACGACGGCAT[illegible]
	Reverse PCR primer	CTGAGGCTCACTGAGACCT
	PCR probe	(SIMA)-ATTCGCACC(T-BHQ1)CGACACGACGGCATTCA
	Forward PCR primer	ACATTCTAAGGCACGCGG
miR-15a-5p	RT primer	CCTGAGTCGTGAACCAGACAGACACAATCGCACCTCGACTCAGGCACAA[illegible]
	Reverse PCR primer	GTGAACCAGACAGACACAA
	PCR probe	(SIMA)-TCGCACC(T-BHQ1)CGACTCAGGCACAAACC
	Forward PCR primer	CAACCATCTAGCAGCACATAAT
miR-182-5p	RT primer	GTCGTGTCTGAGGCTCACTGAGACCTTTCGCACCCTCGACACGACAGTGT[illegible]
	Reverse PCR primer	CTGAGGCTCACTGAGACCT
	PCR probe	(SIMA)-TTCGCACCC(T-BHQ1)CGACACGACAGTGTGAG
	Forward PCR primer	GGACTTTGGCAATGGTAGAA
miR-196b-5p	RT primer	GTCGTGTCTGAGGCTCACTGAGTCCTATTCGCACCTCGACACGACCCCAA[illegible]
	Reverse PCR primer	CTGAGGCTCACTGAGTCCT
	PCR probe	(SIMA)-ATTCGCACC(T-BHQ1)CGACACGACCCCAACAA
	Forward PCR primer	CACCAGCTAGGTAGTTTCCTG
miR-20a-5p	RT primer	GTCGTGTCTGAGGCTCACTGAGACCTATTCGCACCTCGACACGACCTACC[illegible]
	Reverse PCR primer	CTGAGGCTCACTGAGACCT
	PCR probe	(SIMA)-ATTCGCACC(T-BHQ1)CGACACGACCTACCTGC
	Forward PCR primer	CACCAGCTAAAGTGCTTATAGTG

miR-221-3p	RT primer	GTCGTGTCTGAGGCTCACTGAGACCTATTCGCACCTCGACACGACGAAAC
	Reverse PCR primer	CTGAGGCTCACTGAGACCT
	PCR probe	(SIMA)-ATTCGCACC(T-BHQ1)CGACACGACGAAACCCAG
	Forward PCR primer	CAGCAGCTACATTGTCTGC
miR-23b-3p	RT primer	GTCGTGTCTGAGGCTCACTGAGACCTTTCGCACCCTCGACACGACGGAAA
	Reverse PCR primer	CTGAGGCTCACTGAGACCT
	PCR probe	(SIMA)-TTCGCACCC(T-BHQ1)CGACACGACGGAAATCC
	Forward PCR primer	CAGCACATCACATTGCCAG
miR-29b-3p	RT primer	GTCGTGTCTGAGGCTCACTGAGACCTTTCGCACCCTCGACACGACAA(C-L LNA)GAT
	Reverse PCR primer	CTGAGGCTCACTGAGACCT
	PCR probe	(SIMA)-TTCGCACCC(T-BHQ1)CGACACGACAACACTGAT
	Forward PCR primer	CAGCACTAGCACCATTTGAA
miR-30a-5p	RT primer	GTCGTGTCGTGAACCAGACAGACACAATTCGCACCCTCGACACGACCTTC
	Reverse PCR primer	GTGAACCAGACAGACACAA
	PCR probe	(SIMA)-TTCGCACCC(T-BHQ1)CGACACGACCTTCCAGT
	Forward PCR primer	AGGCCTGTAAACATCCTCG
miR-7-5p	RT primer	CTGAGTCGTGAACCAGACAGACACAACCTACGCTCTCCGACTCAGGCAC
	Reverse PCR primer	GTGAACCAGACAGACACAA
	PCR probe	(SIMA)-CCTA(C-LNA)GCTC(T-BHQ1)CCGACTCAGGCACAACAAA
	Forward PCR primer	AGCGTCTGGAAGACTAGTGA
miR-145-5p	RT primer	CGTGTCGCCTTGTAGCACGACCTTATTCGCACCCTCGACACGACAGGGATTC
	Reverse PCR primer	GCCTTGTAGCACGACCTTA
	PCR probe	(R6G)-TTCGCACCC(T-BHQ1)CGACACGACAGGGATTC
	Forward PCR primer	ACACGTCCAGTTTTCCCAG
miR-155-5p	RT primer	GTCAGAGCGCTCTTCTAGCACCACTCTATCCTACCCTCGCTCTGACACCCCTA
	Reverse PCR primer	GCTCTTCTAGCACCACTCTA
	PCR probe	(R6G)-TCCTACCC(T-BHQ1)CGCTCTGACACCCCTA
	Forward PCR primer	CCCAGCTTAATGCTAATCGTGA
let-7a-5p	RT primer	GTCGTGTCTGAGGCTCACTGACACAATTCGCACCCTCGACACGACAACTATAC
	Reverse PCR primer	CTGAGGCTCACTGACACAA
	PCR probe	(R6G)-C+C+C+T+CGA+CACGACAACTATAC-(BHQ1)
	Forward PCR primer	CAGCACTGAGGTAGTAGGTT
miR-16-5p	RT primer	GTCGTGTCTGAGGCTCACTGAGACCTATTCGCACCTCGACACGACCGCCAATA
	Reverse PCR primer	CTGAGGCTCACTGAGACCT
	PCR probe	(R6G)-ATTCGCACC(T-BHQ1)CGACACGACCGCCAATA
	Forward PCR primer	CCAGCTAGCAGCACGTAAA

miR-181a-5p	RT primer	GTCGTGTCTGAGGCTCACTGAGACCTATTCGCACCTCGACACGACACCCACCG
	Reverse PCR primer	CTGAGGCTCACTGAGACCT
	PCR probe	(R6G)-ATTCGCACC(T-BHQ1)CGACACGACACCCACCG
	Forward PCR primer	CCAGCAACATTCATTGCTGT
miR-191-5p	RT primer	GTCGTGTCTGAGGCTCACTGAGACCTATTCGCACCTCGACACGACCAGCTGCT
	Reverse PCR primer	CTGAGGCTCACTGAGACCT
	PCR probe	(R6G)-ATTCGCACC(T-BHQ1)CGACACGACCAGCTGCT
	Forward PCR primer	CAGCCAACGGAATCCCAAA
miR-26a-5p	RT primer	CGTGATGCGCCTTGTAGCACGACCTTATTCGCACCCTCGCATCACGAGCCTATC
	Reverse PCR primer	GCCTTGTAGCACGACCTTA
	PCR probe	(R6G)-TTCGCACCC(T-BHQ1)CGCATCACGAGCCTATC
	Forward PCR primer	CGAGCCATTCAAGTAATCCAG
miR-378-3p	RT primer	GTCGTGTCTGAGGCTCACTGAGACCTATTCGCACCTCGACACGACGCCTTCTG
	Reverse PCR primer	CTGAGGCTCACTGAGACCT
	PCR probe	(R6G)-ATTCGCACC(T-BHQ1)CGACACGACGCCTTCTG
	Forward PCR primer	CTGAGGCTCACTGAGACCT

(+C=C-LNA; +T=T-LNA; R6G -Rhodamine 6G (Rhodamine 590); BHQ1 - Black Hole Quencher-1; LNA - Locked Nucleic Acid).
